# Drug repositioning based on individual bi-random walks on a heterogeneous network

**DOI:** 10.1186/s12859-019-3117-6

**Published:** 2019-12-24

**Authors:** Yuehui Wang, Maozu Guo, Yazhou Ren, Lianyin Jia, Guoxian Yu

**Affiliations:** 1 College of Computer and Information Sciences, Southwest University, Beibei, Chongqing, 400715 China; 2 School of Electrical and Information Engineering, Beijing University of Civil Engineering and Architecture, Beijing, 100044 China; 3 School of Computer Science and Engineering, University of Electronic Science and Technology of China, Chengdu, 611731 China; 4 College of Information Engineering and Automation, Kunming University of Science and Technology, Kunming, 650504 China; 5 Hubei Key Laboratory of Intelligent Geo-Information Processing, China University of Geosciences, Wuhan, 430074 China

**Keywords:** Drug repositioning, Drug-disease heterogeneous network, Individual walk-length, Bi-random walks

## Abstract

**Background:**

Traditional drug research and development is high cost, time-consuming and risky. Computationally identifying new indications for existing drugs, referred as *drug repositioning*, greatly reduces the cost and attracts ever-increasing research interests. Many network-based methods have been proposed for drug repositioning and most of them apply random walk on a heterogeneous network consisted with disease and drug nodes. However, these methods generally adopt the same walk-length for all nodes, and ignore the different contributions of different nodes.

**Results:**

In this study, we propose a drug repositioning approach based on individual bi-random walks (DR-IBRW) on the heterogeneous network. DR-IBRW firstly quantifies the individual work-length of random walks for each node based on the network topology and knowledge that similar drugs tend to be associated with similar diseases. To account for the inner structural difference of the heterogeneous network, it performs bi-random walks with the quantified walk-lengths, and thus to identify new indications for approved drugs. Empirical study on public datasets shows that DR-IBRW achieves a much better drug repositioning performance than other related competitive methods.

**Conclusions:**

Using individual random walk-lengths for different nodes of heterogeneous network indeed boosts the repositioning performance. DR-IBRW can be easily generalized to prioritize links between nodes of a network.

## Background

Traditional drug research and development depends on cell-based or target-based screening of chemical compounds to identify a small subset of ‘hits’. The identification process aims to further increase their affinity, efficacy and selectivity, before moving forward to animal tests and clinical trials [1]. Drug development in general is complicated, time-consuming and expensive with high-risk [2]. In light of these difficulties in traditional drug discovery, identifying new indications for existing drugs, also known as *drug repositioning*, has attracted increasing interests from both the pharmaceutical industry and research community [3]. Drug repositioning is much more economic compared with traditional approaches, it offers a promising alternative to reduce the cost and time, since the repositioned drug has already passed the required safety tests.

However, most successfully repositioned drugs up to date have been the consequence of incidental observations of unexpected efficacy and side effects in the development or on the market [4]. For example, Sildenafil was originally tested for angina, now is indicated for erectile dysfunction and pulmonary hypertension [2]; Minoxidil was originally tested for hypertension; now is indicated for hair loss [5]. With the influx of big biochemical and phenotypic data, drug repositioning holds great potential for precise medicine. It is profitable and promising to develop computational methods to predict new indications for approved drugs on large scale.

Some computational drug repositioning methods have been proposed and they can be roughly divided into two categories: focusing on the interactions between drugs and the targets; and focusing on exploiting the knowledge of diseases and drugs [6]. To name a few, Bleakley and Yamanishi [7] developed a bipartite local model (BLM) to predict target proteins of a given drug and target drugs of a give protein, and then combine these two predictions to give a final prediction for each candidate drug-target interaction. Cheng et al. [8] used a drug-target bipartite network topology similarity and a network based inference algorithm (NBI) to infer new targets for known drugs. Wang et al. [9] used known drug-target interactions as well as drug-drug and target-target similarities to construct a heterogeneous network, and then introduced a Heterogeneous Graph Based Inference (HGBI) method to iteratively update the strength between unlinked drug-target pairs based on all the paths in the network connecting them. These drug-target prediction methods can be readily adopted for drug repositioning.

Chiang et al. [10] attempted to predict novel associations between drugs and diseases based on the widely-adopted ‘guilt-by-association’ principle. This principle assumes that if a drug can treat one of two similar diseases, then it might treat the other also; alternatively a disease can be treated by two similar drugs. Following this principle, Gottlieb et al. [11] measured the similarity between the pertaining drug and disease of drug-disease pairs that are known to be associated based on multiple drug-drug sources and disease-disease similarity metrics, and then ranked the accumulative evidence for association using a logistic regression scheme to predict novel drug indications. Wang et al. [1] integrated omics data about diseases, drugs and drug targets to construct a heterogeneous network and then applied random walks on the network to replenish missing associations between drugs and diseases. Martinez et al. [6] integrated information on diseases, drugs and targets (proteins) to construct a heterogeneous network and then performed propagation flow on the network to prioritize candidate associations between diseases and drugs according to their interconnections in the network. Luo et al. [12] proposed MBiRW to predict drug-disease associations. MBiRW employs known drug-disease associations to improve the drug-drug and disease-disease similarity measures; and then integrates the similarity networks and drug-disease associations to build a drug-disease heterogeneous network; after that, it performs bi-random walk with restart on the network to predict novel potential drug-disease associations. Liu et al. [13] performed a drug-centric random walk and a disease-centric random walk to obtain the association confidence between the disease nodes and drug nodes of a heterogeneous network.

Most of these aforementioned methods in essence are random walk based solutions. Although they make use of the network topology from different perspectives, they ignore the different contributions of different nodes on transferring the information on the network and almost all adopt a *fixed walk-length* for all nodes. To overcome this issue, we propose a novel drug repositioning approach (called DR-IBRW) that performs bi-random walk with restart on a heterogeneous network with quantified individual walk-length for each node. DR-IBRW uses disease symptom information [14] and drug chemical fingerprints [15] to construct a composite disease-disease similarity network, drug-drug similarity network. It then quantifies the individual walk-length for each node based on the topology of known drug-disease association network. Next, it constructs a heterogeneous network based on these three networks. After that, it performs bi-random walks with the quantified walk-lengths to account for the structural differences of these networks and contribution differences of different nodes (including diseases and drugs), and to predict new associations between drugs and diseases, and thus to accomplish the drug repositioning. We evaluate and compare the performance of DR-IBRW on several public datasets. DR-IBRW obtains much better performance than other related comparing methods [7–9, 12, 16] in identifying new indications for existing drugs, and the quantified individual walk-length indeed contributes to an improved prediction performance. We want to remark that the proposed individual bi-random walk solution is *different* from existing personalized random walk solutions [17, 18] that mainly focus on setting different restart probabilities for different nodes.

## Materials and methods

### Dataset

The datasets used in this work include drug-disease associations, drug fingerprints and disease symptoms. We collected 4219 diseases from MeSH [19] and 322 symptoms for each disease from the supplementary material of [14]. The drug-disease association dataset was obtained from [20], it includes 3250 known drug-disease associations involving 799 drugs and 719 diseases. We also collected 881 fingerprints for each drug from PubChem [15]. Since only 525 diseases can find their relevant symptom information from the supplementary material of [14], the final processed dataset includes 525 diseases, 718 drugs and 2177 drug-disease associations. All these data were collected on November 1st, 2017.

### Similarity measures

We separately apply a four-step measurement to quantify the inner-similarity between diseases and between drugs. The first three steps are based on the comprehensive similarity measurement used by Luo et al. [12]. In the fourth step, we use Gaussian interaction profile kernel similarity [21] to measure the similarity between drugs and diseases. Finally, we combine these similarities to form the composite similarity between diseases and between drugs. The four-step procedure of measuring the similarity between drugs is briefly introduced as follows.

*Step 1:* Based on the chemical fingerprints of the drug molecules, we can initially measure the similarity $\mathbf {S}_{r}^{1} \in \mathbb {R}^{n_{r} \times n_{r}}$ between *n*_*r*_ drugs via the widely used Cosine similarity metric [22]. Let **r**_*i*_ and **r**_*j*_ be the vector forms of the chemical fingerprints of drug *r*_*i*_ and *r*_*j*_, the chemical similarity $\mathbf {S}_{r}^{1}(r_{i},r_{j})$ between drug *r*_*i*_ and *r*_*j*_ is defined as:

1 $$ \mathbf{S}_{r}^{1}(r_{i},r_{j}) = \frac{\mathbf{r}_{i}^{T} \mathbf{r}_{j}}{\left \| \mathbf{r}_{i} \right \|\left \|\mathbf{r}_{j} \right\|},  $$

*Step 2:* Too small similarity provides little information for drug repositioning and can be transformed into zeros for accurate prediction [9, 12]. We partition $\mathbf {S}_{r}^{1}$ into ten subranges ((0,0.1], (0.1,0.2], etc.) and calculate the average similarity of drug pairs with shared diseases for each subrange. We also randomly shuffle $\mathbf {S}_{r}^{1}$ and repeat the partition and calculation process again. If the average of the non-shuffled subrange is smaller than that of the respective shuffled subrange, the drug similarities divided into this subrange are viewed as not informative; otherwise, they are informative. We then adopt a logistic function [23] to shrink these non-informative similarities to zero and to enlarge these informative similarities. The logistic function is defined as follows:

2 $$ \mathbf{S}_{r}^{2}(r_{i},r_{j})=\frac{1}{1 + e^{c\mathbf{S}_{r}^{1}(r_{i},r_{j}) + d}},  $$

where *c* and *d* are the parameters can be tuned to control the adjustment of $\mathbf {S}_{r}^{1}$. *c* is the upper bound of the first subrange whose average similarity is smaller than that of the respective shuffled subrange, *d*=*log*(999). After that, we obtain a updated drug similarity matrix $\mathbf {S}_{r}^{2}$.

*Step 3:* Two drugs are more similar if they are grouped into the same cluster. To make use of this assumption, we first construct a new weighted drug sharing network with drugs as nodes and edge weight reflecting the number of common diseases by respective pair nodes. After that, we adopt a graph clustering method, ClusterONE [24], to identify potential drug clusters on the network. We then add the clustering cohesiveness of a cluster with $\mathbf {S}_{r}^{2}$ if and only if the two drugs belong to that cluster.

3 $$ f(\mathcal{C})=\frac{W_{in}(\mathcal{C})}{(W_{in}(\mathcal{C})+W_{bound}(\mathcal{C})+P(\mathcal{C}))},  $$

where $W_{in}(\mathcal {C})$ denotes the total weight of edges within a cluster of vertices, $W_{bound}(\mathcal {C})$ represents the total weight of edges connecting nodes of this cluster to nodes of other clusters, and $P(\mathcal {C})$ is the penalty term. Suppose that drug *r*_*i*_ and drug *r*_*j*_ locating in the same cluster $\mathcal {C}$, the comprehensive drug similarity $\mathbf {S}_{r}^{3}(r_{i},r_{j})$ between drug *r*_*i*_ and *r*_*j*_ is defined as $(1+f(\mathcal {C}))*\mathbf {S}_{r}^{2}(r_{i},r_{j})$. In this way, we obtain an improved drug similarity matrix $\mathbf {S}_{r}^{3}$.

*Step 4:* Based on the assumption that similar drugs tend to show similar interaction and non-interaction profiles with the diseases, we further use Gaussian interaction profile kernel similarity to measure the similarity between drugs [21, 25, 26]. The interaction profile *IP*(*r*_*i*_) of drug *r*_*i*_ is defined as a binary vector encoding the presence or absence of the known associations between the drug and *n*_*d*_ diseases. The Gaussian interaction profile kernel similarity between two drugs (*r*_*i*_ and *r*_*j*_) is computed as follows:

4 $$ \mathbf{S}_{r}^{KR}(r_{i},r_{j})=exp\left({-\Upsilon_{r}}{\|IP(r_{i})-IP(r_{j})\|^{2}}\right),  $$

5 $$ \Upsilon_{r}=\tilde{\Upsilon}_{r}/\left(\frac{1}{n_{r}}\sum_{i=1}^{n_{r}}\|IP(r_{i})\|^{2}\right),  $$

where *Υ*_*r*_ is the kernel bandwidth, $\tilde {\Upsilon }_{r}$ is the average number of associated diseases per drug.

To this end, we combine $\mathbf {S}_{r}^{3}$ and $\mathbf {S}_{r}^{KR}$ into the composite similarity matrix **S**_*r*_ between *n*_*r*_ drugs as follows:

6 $$ \mathbf{S}_{r}=\left(\mathbf{S}_{r}^{3}+ \mathbf{S}_{r}^{KR}\right)/2  $$

Following the above four-step, we can also compute the composite similarity $\mathbf {S}_{d} \in \mathbb {R}^{n_{d} \times n_{d}}$ between *n*_*d*_ diseases based on the symptom information of these diseases and drug-disease associations.

### Quantifying individual walk-length

Network-based drug repositioning methods generally apply random walk on a network with a fixed walk-length for all nodes to explore the network topology [12, 27, 28]. They ignore the different contributions of different nodes to some extent. Given that, we introduce an individual walk-length measure and try to make better use of the topology of known drug-disease association bipartite network $\mathbf {W}_{rd} \in \mathbb {R}^{n_{r} \times n_{d}}$ of *n*_*r*_ drugs and *n*_*d*_ diseases. **W**_*rd*_(*r*_*i*_,*d*_*j*_)=1 if the association between the drug *r*_*i*_ and disease *d*_*j*_ is known; and 0 otherwise.

The walk-length of a node generally depends on its influence in the network [29]. We extend the Jaccard index measure introduced by Lu et al. [16] to quantify the individual walk-length of nodes. Suppose $\mathcal {N}^{r}(r_{i})$ denote the set of neighbours of drug *r*_*i*_ and $\mathcal {N}^{d}(d_{j})$ denote the set of neighbours of disease *d*_*j*_, if *r*_*i*_ and *d*_*j*_ share many common neighbours, they will be more probably influenced with each other. For a randomly selected feature *f* of either *r*_*i*_ or *d*_*j*_, traditional Jaccard index measures the probability that both *r*_*i*_ and *d*_*j*_ have that feature as follows [30]:

7 $$ \mathbf{JI}^{\prime}(r_{i},d_{j})=\frac{|{\mathcal{N}^{r}(r_{i})\cap\mathcal{N}^{d}(d_{j})}|}{|{\mathcal{N}^{r}(r_{i})\cup\mathcal{N}^{d}(d_{j})}|},  $$

Since there is no relationship between diseases or between drugs in the drug-disease bipartite network, $\mathcal {N}^{r}(r_{i})\cap \mathcal {N}^{d}(d_{j})$ is an empty set. For this reason, we have to modify the definition of Jaccard index for a bipartite graph. Particularly, we define $\widehat {\mathcal {N}}^{r}(r_{i})=\cup _{c\in \mathcal {N}^{r}(r_{i})}\mathcal {N}^{d}(c)$ as the set of drugs associated with *r*_*i*_’s neighbours. Then the Jaccard index on the bipartite network is defined as follows:

8 $$ \mathbf{JI}(r_{i},d_{j})=\frac{|{\mathcal{N}^{r}(r_{i})\cap\widehat{\mathcal{N}}^{d}(d_{j})}|}{|{\mathcal{N}^{r}(r_{i})\cup\widehat{\mathcal{N}}^{d}(d_{j})}|},  $$

**JI**(*r*_*i*_,*d*_*i*_) represents the influence between drug-disease pair (*r*_*i*_,*d*_*j*_). We assume that a node with *high* quantified influence has *more* probability to interact with others during the random walk process, and this node should have *larger* walk-length. Based on this assumption, we can measure the walk-length of each node as follows:

9 $$ \mathbf{L}_{r}(r_{i})=\sum_{j=1}^{n_{d}} \mathbf{JI}(r_{i},d_{j}), \quad \mathbf{L}_{d}(d_{j})=\sum_{i=1}^{n_{r}} \mathbf{JI}(r_{i},d_{j}),  $$

where $\mathbf {L}_{r}\in \mathbb {R}^{n_{r}}$ and $\mathbf {L}_{d}\in \mathbb {R}^{n_{d}}$ store the individual walk-lengths of *n*_*r*_ drugs and *n*_*d*_ diseases, respectively.

### Individual bi-random walk

**Fig. 1:**
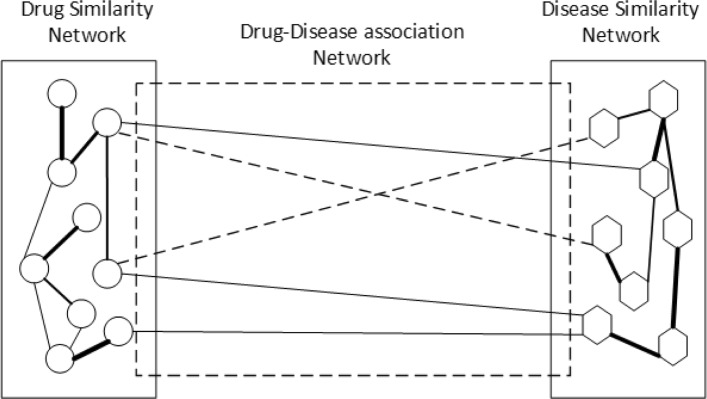
A heterogeneous network consists of drug similarity network $\mathbf {S}_{r} \in \mathbb {R}^{n_{r} \times n_{r}}$ with *n*_*r*_ drugs, disease similarity network $\mathbf {S}_{d} \in \mathbb {R}^{n_{d} \times n_{d}}$ with *n*_*d*_ diseases, drug-disease association network $\mathbf {W}_{rd} \in \mathbb {R}^{n_{r} \times n_{d}}$ between *n*_*r*_ drugs and *n*_*d*_ diseases. Each circle represents a drug, each hexagon represents a disease. In the drug (disease) similarity network, the solid edges describe the similarities of drug (disease) pairs. In the drug-disease association network, the solid edges indicate the known drug-disease associations, and the dashed edges indicate the potential associations between drugs and diseases, which are the new indications of drugs

Based on the inner similarity network (defined by **S**^*r*^) of drugs, the inner similarity network (defined by **S**^*d*^) of diseases, and the drug-disease bipartite network initialized by known drug-disease associations, we can construct a heterogeneous network of drugs and diseases (see Fig. 1 for example). We adopt a bi-random walk with restart procedure [27] on the heterogeneous network. Compared with traditional random walk with restart, the bi-random walk with restart can separately propagate information in different subnetworks, instead of the global network [28]. For this reason, bi-random walk can separately account for the inner structure of disease similarity network and of drug similarity network, and also make use of associations between drugs and diseases.

A random walker can take a drug as the starting node, its associated diseases as intermediate nodes, and then traverse to other disease nodes. In this way, we can get probabilistic associations between the drug and new diseases, and thus identify potential new indications of the drug. To mimic this process, we perform random walk with restart starting from drug nodes and then traversing to disease nodes based on the quantified individual walk-length and the heterogeneous network topology as follows:

10 $$\begin{array}{@{}rcl@{}} \mathbf{F}_{r}^{t}(r_{i},d_{j}) =\begin{cases} \alpha \sum_{k=1}^{n_{d}}\tilde{\mathbf{S}}_{d}(d_{k},d_{j})\mathbf{F}_{r}^{t-1}(r_{i},d_{k})+ \\ (1-\alpha)\mathbf{W}_{rd}(r_{i},d_{j}), \text{if} \ t \leq \lfloor \mathbf{L}_{r}(r_{i})\rfloor \\ \mathbf{F}_{r}^{t-1}(r_{i},d_{j}), \quad \text{otherwise} \end{cases} \end{array} $$

where $\mathbf {F}_{r}^{t}(r_{i},d_{j})$ is the predicted relevance between drug *r*_*i*_ and disease *d*_*j*_ in the *t*-th iteration, $\mathbf {F}_{r}^{0}=\mathbf {W}_{rd}$, *α*>0 controls the probability for a walker staying at the starting point, $\tilde {\mathbf {S}}_{d}=\mathbf {D}_{d}^{-\frac {1}{2}}*\mathbf {S}_{d}*\mathbf {D}_{d}^{-\frac {1}{2}}$ is the Laplacian normalized result of **S**_*d*_ and **D**_*d*_ is a diagonal matrix with $\mathbf {D}_{d}(d_{j},d_{j})=\Sigma _{k=1}^{n_{d}}\mathbf {S}_{d}(d_{j},d_{k})$. If *t*>**L**_*r*_(*r*_*i*_), the random walker starting from *r*_*i*_ will not jump any more. We want to recomment that unlike traditional random walks and bi-random walks that adopt the same walk-length for all the nodes, the walk-length of a node in Eq. (10) is adaptively set based on its topology relationship with other nodes and is different from the walk-lengths of other nodes.

Similarly, a random walker can also start from a disease node and then traverse to drug nodes based on known drug-disease relationships and drug similarity network. In this way, we can obtain another probability between the disease and drug. To simulate this process, we perform random walk with restart from the disease node (*d*_*j*_) as follows:

11 $$\begin{array}{@{}rcl@{}} \mathbf{F}_{d}^{t}(r_{i},d_{j}) =\begin{cases} \alpha \sum_{k=1}^{n_{r}}\tilde{\mathbf{S}}_{r}(r_{i},r_{k})\mathbf{F}_{d}^{t-1}(r_{k},d_{j})+ \\ (1-\alpha)\mathbf{W}_{rd}(r_{i},d_{j}), \text{if} \ t \leq \lfloor\mathbf{L}_{d}(d_{j})\rfloor\\ \mathbf{F}_{d}^{t-1}(r_{i},d_{j}), \quad \text{otherwise} \end{cases} \end{array} $$

where $\mathbf {F}_{d}^{t}(r_{i},d_{j})$ is the predicted relevance between drug *r*_*i*_ and disease *d*_*j*_ in the *t*-th iteration, and the same normalization procedure is applied to **S**_*r*_ to construct the normalization matrix $\tilde {\mathbf {S}}_{r}=\mathbf {D}_{r}^{-\frac {1}{2}}*\mathbf {S}_{r}*\mathbf {D}_{r}^{-\frac {1}{2}}$, **D**_*r*_ is a diagonal matrix with $\mathbf {D}_{r}(r_{i},r_{i})=\Sigma _{j=1}^{n_{r}}\mathbf {S}_{r}(r_{i},r_{j})$.

After iteratively applying Eqs. (10-11) with individual walk-lengths, we can obtain **F**_*r*_ and **F**_*d*_, which separately reflect the association confidences between *n*_*r*_ drugs and *n*_*d*_ diseases from the perspective of the disease similarity network, and from the drug similarity network, along with the known drug-disease associations. To this end, we integrate them as follows:

12 $$ \mathbf{F} = \frac{\mathbf{F}_{r}+\mathbf{F}_{d}}{2}  $$

Obviously, the larger the value of **F**(*r*_*i*_,*d*_*j*_), the larger the probability that drug *r*_*i*_ associated with disease *d*_*j*_ is. In this way, we can finally identify new indications for existing drugs. The whole procedure of DR-IBRW is described in Algorithm 1.


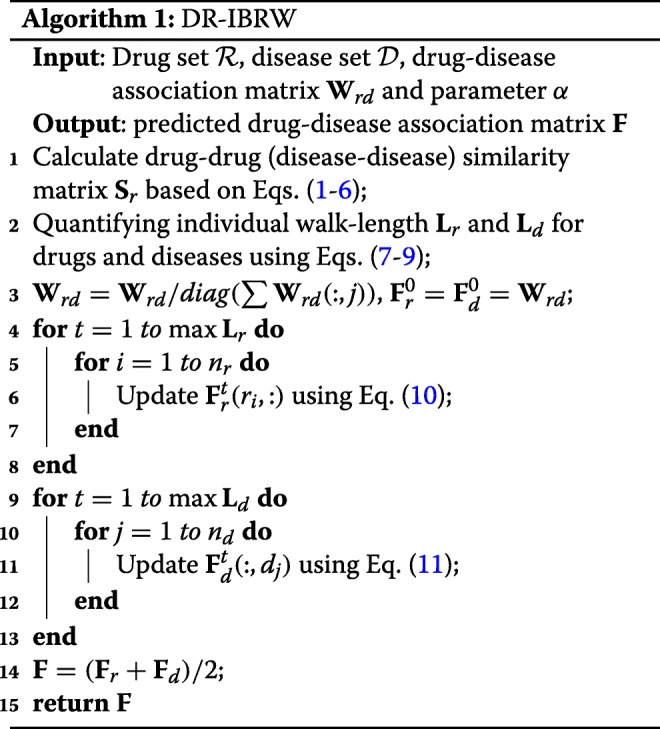


## Results and discussion

### Performance comparison with other methods

**Fig. 2:**
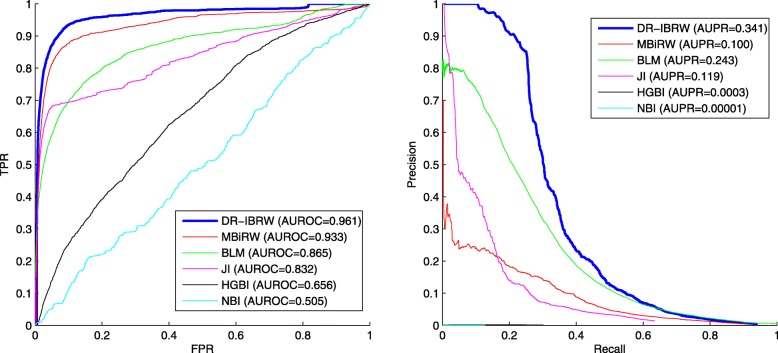
The ROC and PR curves of DR-IBRW and comparison algorithms. AUROC and AUPR are the values of area under the ROC and PR curve, respectively

DR-IBRW is compared with five related and recent methods (MBiRW [12], BLM [7], JI (Jaccard Index) [16], HGBI [9] and NBI [8]) on the processed dataset. MBiRW, BLM, HGBI and NBI were introduced in the Introduction, the last four methods are originally developed for predicting drug-target interactions and can be directly adopted to predict drug-disease associations. Parameters of these comparing methods are set (or optimized) as the authors suggested (or provided) in their respective papers or codes. As to DR-IBRW, *α* for random walk restart probability is set to 0.1. To reach a comprehensive evaluation, we use six widely used metrics, namely *AUROC*, *AUPR*, *Macro-F1*, *Micro-F1*, *Precision*, *Recall*. These metrics are also used by those comparing methods [7–9, 12, 16]. The formal definitions of these metrics are omitted here, but interested readers than can find the formal definitions of these metrics in these references and references therein. All these methods follow ten fold cross-validation experimental protocol, and then report the average results and standard deviation in Table 1. In addition, we also plot the receiver operating characteristic (ROC) curve and precision recall (PR) curve, and the value of area under perspective curve in Fig. 2.

**Table 1:** The results of DR-IBRW and five comparing methods

Methods	Micro-F1	Macro-F1	Precision	Recall
DR-IBRW	0.395 ±0.002	**0.328±0.001**	0.212 ±0.000	**0.766±0.007**
MBiRW	0.294 ±0.004	0.245 ±0.003	0.158 ±0.001	0.572 ±0.016
BLM	**0.413±0.006**	0.304 ±0.002	**0.224±0.003**	0.740 ±0.002
JI	0.229 ±0.001	0.188 ±0.001	0.123 ±0.000	0.427 ±0.002
HGBI	0.013 ±0.000	0.010 ±0.000	0.007 ±0.000	0.021 ±0.000
NBI	0.009 ±0.000	0.007 ±0.000	0.004 ±0.000	0.016 ±0.000

The entry in boldface represent the method perform best in this evaluation metric

We can easily find that DR-IBRW achieves better performance than these comparing methods. Although both DR-IBRW and MBiRW utilize the drug similarity network, disease similarity network and drug-disease association network to construct a heterogeneous network, and then apply bi-random walks with restart to account for the structural difference of this network, DR-IBRW still performs significantly better than MBiRW. That is because DR-IBRW takes into account the different contributions of different nodes and applies individual walk-lengths for them, whereas MBiRW equally treats all nodes and applies the same walk-length. In addition, DR-IBRW uses the Gaussian interaction profile kernel similarity to strengthen the effect of known drug-disease associations.

HGBI also applies random walks with restart on the heterogeneous network, but it does not take into account structural difference between drug similarity network and disease similarity network. BLM tries to build a separate classifier for each drug and each drug, but it is still suffered from biased training data, since there are more negative samples than positive samples (known associations). In fact, a number of negative samples should be positive ones. For this reason, BLM has a high Precision and Recall but with a low AUPR value. JI takes into account the influence of a node in the bipartite network and uses common neighbours to predict drug-disease associations. NBI only utilizes known drug-disease associations to run a two-step diffusion model on the bipartite graph and it can not predict new associations for a drug without known associations. For these reasons, both JI and NBI are outperformed by DR-IBRW.

### Individual walk-length analysis

**Fig. 3:**
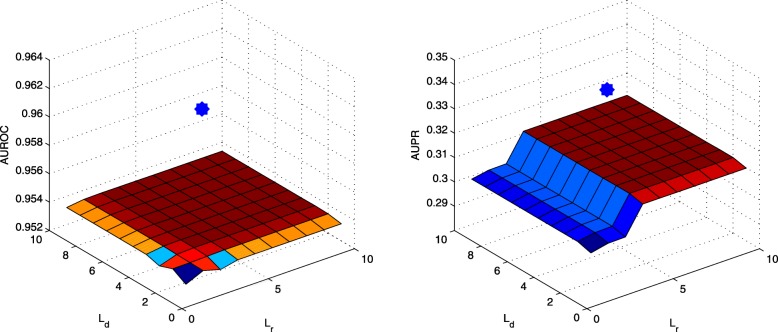
The AUROC and AUPR values of DR-IBRW with different fixed walk-lengths. The blue star is the value of DR-IBRW with individual walk-lengths

To study the contribution of our proposed individual walk-lengths, we also test the performance of DR-IBRW with fixed walk-lengths for all the nodes by varying walk-length in the disease network and drug network from 0 to 10, respectively. Fig. 3 reveals the AUROC and AUPR of DR-IBRW under different combined configurations of **L**_*r*_ and **L**_*d*_. From this figure, we can clearly see that the AUROC stops increasing when **L**_*r*_ and **L**_*d*_ are larger than 2, and the AUROC and AUPR values with a fixed walk-length are smaller than those of DR-IBRW with individual walk-lengths. This comparison further corroborates the effectiveness and rationality of individual walk-lengths.

### Drug and disease similarity analysis

**Fig. 4:**
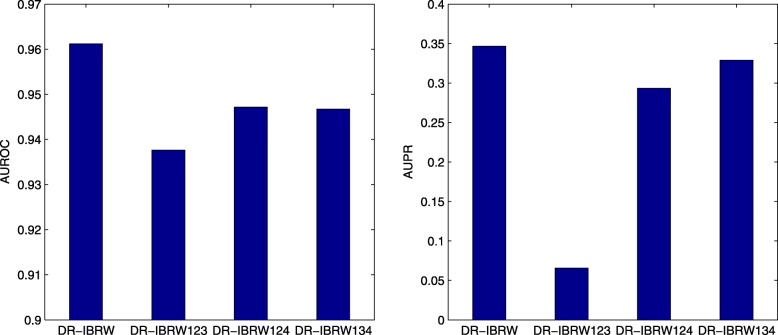
The AUROC and AUPR values of DR-IBRW and its variants

As introduced in Section 5, we measure the composite inner similarity between diseases and drugs in four steps. To investigate the impact of these four steps and the contribution of Gaussian interaction kernel profile similarity, we introduce three variants (DR-IBRW123, DR-IBRW124, DR-IBRW134) of DR-IBRW. Particularly, DR-IBRW123 only uses the first three steps (as done by Luo et al. [12]), or excludes the Gaussian interaction kernel profile similarity, to measure the inner similarity between diseases and between drugs. Similarly, DR-IBRW134 excludes the second step without shrinking low similarity and enlarging high similarity. DR-IBRW124 follows the same naming rule. The AUROC and AUPR values of DR-IBRW and its variants by ten fold cross-validations are shown in Fig. 4. Obviously, the AUROC and AUPR values of DR-IBRW123 are lower than those of other methods, which show the contribution of Gaussian interaction profile kernel similarity for drug repositioning. Another interesting observation is that DR-IBRW134 has a higher AUPR value than other variants and DR-IBRW. The cause is that AUPR and AUROC measure the performance from different perspectives and under varying thresholds. The second step may wrongly shrink low similarity and enlarge high similarity, and thus compromise the performance.

### Experiments on another two datasets

We collected another two datasets to further study the performance of DR-IBRW. The first dataset (named ‘Gottlieb’s Dataset’), was obtained from [11]. This dataset contains 1933 known drug-disease associations involving 593 drugs registered in DrugBank and 313 diseases listed in the Online Mendelian Inheritance in Man (OMIM). The another dataset (‘Luo’s Dataset’) is obtained from [12], it includes 663 drugs registered in DrugBank, 409 diseases listed in OMIM database and 2352 known drug-disease associations. Table 2 reports the results of 10 fold cross-validation of DR-IBRW and comparing methods on these two datasets. The experimental setups are kept the same as in previous experiments. From these tables, we can also find that DR-IBRW again obtains much better performance than these comparing methods across different evaluation metrics.

**Table 2:** The performance results of DR-IBRW and comparing methods on Gottlieb’s dataset [11] and Luo’s dataset [12]

	AUROC	AUPR	Micro-F1	Macro-F1	Precision	Recall
	Gottlieb’s dataset					
DR-IBRW	**0.955±0.000**	**0.499±0.174**	**0.613±0.006**	**0.513±0.005**	**0.332±0.002**	0.880 ±0.000
MBiRW	0.933 ±0.000	0.213 ±0.028	0.294 ±0.004	0.244 ±0.003	0.256 ±0.001	**0.906±0.000**
BLM	0.865 ±0.000	0.298 ±0.003	0.583 ±0.001	0.479 ±0.001	0.315 ±0.000	0.891 ±0.000
JI	0.845 ±0.001	0.247 ±0.043	0.385 ±0.003	0.462 ±0.004	0.250 ±0.001	0.894 ±0.181
HGBI	0.811 ±0.000	0.016 ±0.000	0.187 ±0.001	0.157 ±0.001	0.101 ±0.000	0.367 ±0.007
NBI	0.503 ±0.000	0.000 ±0.000	0.022 ±0.000	0.018 ±0.000	0.012 ±0.000	0.039 ±0.001
	Luo’s dataset					
DR-IBRW	**0.964±0.000**	**0.529±0.167**	**0.537±0.006**	0.452 ±0.004	**0.294±0.002**	**0.895±0.002**
MBiRW	0.945 ±0.000	0.285 ±0.042	0.431 ±0.004	0.363 ±0.003	0.236 ±0.001	0.835 ±0.013
BLM	0.892 ±0.000	0.424 ±0.017	0.527 ±0.003	**0.463±0.004**	0.278 ±0.001	0.843 ±0.000
JI	0.865 ±0.000	0.287 ±0.041	0.537 ±0.004	0.447 ±0.003	0.294 ±0.001	0.783 ±0.000
HGBI	0.848 ±0.000	0.037 ±0.001	0.170 ±0.001	0.141 ±0.001	0.093 ±0.000	0.318 ±0.005
NBI	0.479 ±0.000	0.000 ±0.000	0.020 ±0.000	0.016 ±0.000	0.011 ±0.000	0.032 ±0.000

The entry in boldface represent the method perform best in this evaluation metric

### Case study

To further demonstrate that the drug-disease associations predicted by DR-IBRW can be confirmed by biological experiments, we apply DR-IBRW to prioritize potential drug-disease pairs. Here, we use all the collected drug-disease associations as training samples, and then select the top 10 drug-disease pairs with the largest association probabilities as the predicted drug-disease associations. After that, we manually check these associations by referring to the associations stored in Comparative Toxicogenomics Database(CTD) [31]. Particularly, we use the data of chemical-disease associations labeled with therapeutic downloaded from CTD. The label therapeutic represents a chemical that has a known or potential therapeutic role in a disease. For the predicted associations cannot find in the CTD, we further manually check them on PubMed and list the supportive PubMed IDs. We highlight the drug-disease associations supported by recent papers in PubMed but not included in CTD in **boldface**. The currently supported and un-supported associations are listed in Table 3.

**Table 3:** DR-IBRW predicted drug-disease associations (top 10 in ranking list), and the corresponding evidence

Drug	Disease	Evidence (PMID)	Rank
Labetalol	Hypertension	6124264; 25692529	1
Irbesartan	Heart Failure	19001508	2
Enalapril	Hypertension	2994986	3
Flurandrenolide	Scalp Dermatoses	without evidence	4
Hydralazine	Hypertension	20687078; 22071816	5
Fenoldopam	Hypertension	8105829	6
Captopril	Hypertension	**6754186; 3520132; 1747216; 23161035**	7
Erythrityl Tetranitrate	Hypertension	without evidence	8
Nitroprusside	Hypertension	21272230; 9796241	9
Ranolazine	Hypertension	**24464752; 26401256**	10

The entries in boldface represent the drug-disease associations supported by recent papers in PubMed but not included in CTD

From Table 3, 6 out of top 10 predicted associations are supported by associations in CTD, the other two drug-disease pairs are supported by recent papers in PubMed but not included in CTD. For instance, Labetalol is an effective agent in essential hypertension as documented in open studies and controlled studies [32]. For another instance, Greminger et al. confirmed the high efficacy of captopril in treatment of severe hypertension refractory to conventional drugs [33]. Meanwhile, ranolazine therapy is safe and well tolerated in a pilot study involving pulmonary arterial hypertension [34]. Although we can not find the direct evidence for the associations of flurandrenolide and scalp dermatoses, flurandrenolide topical is used to treat the itching, redness, dryness, crusting, scaling, inflammation, and discomfort of various skin conditions [35].

These predicted results confirm the capability of DR-IBRW in identifying novel drug-disease associations with high confidence. We want to remark that the 2 unsupported associations should not be viewed as incorrect associations. As more experimental evidence becomes available, they maybe further supported.

We also report the top 10 repositioned examples made by other comparing methods, and then manually check these examples by referring to the associations stored in CTD. We further check the associations that cannot find in the CTD on PubMed and list the supportive PubMed IDs. We highlight the drug-disease associations supported by recent papers in PubMed but not included in CTD in **boldface**. Tables 4, 5, 6, 7 and 8 list the currently supported and un-supported associations for MBiRW, BLM, JI, HGBI and NBI, respectively.

**Table 4:** MBiRW predicted drug-disease associations (top 10 in ranking list), and the corresponding evidence.

Drug	Disease	Evidence (PMID)	Rank
Echothiophate	Esotropia	**13907355; 7166393**	1
Cysteamine	Cystinosis	22532830;23651769	2
Ethanol	Complex Regional Pain Syndromes	without evidence	3
Ethanol	Warts	**9557098**	4
Foscarnet	Herpes Genitalis	without evidence	5
Ribavirin	Respiratory Syncytial Virus Infections	11781627	6
Foscarnet	Cytomegalovirus Infecti	11362300;11050094;10795660	7
Nitisinone	Tyrosinemias	11488774	8
Hydroxocobalamin	Alcoholic Neuropathy	without evidence	9
Methimazole	Goiter	14723259	10

The entries in boldface represent the drug-disease associations supported by recent papers in PubMed but not included in CTD

**Table 5:** BLM predicted drug-disease associations (top 10 in ranking list), and the corresponding evidence

Drug	Disease	Evidence (PMID)	Rank
Cefixime	Streptococcal Infections	2041146	1
Cefdinir	Urinary Tract Infections	**20573040**	2
Ceftibuten	Soft Tissue Infections	without evidence	3
Ceftibuten	Klebsiella Infections	**25813819**	4
Ceftibuten	Urinary Tract Infections	**11605809;2391749**	5
Cefdinir	Soft Tissue Infections	**15313534;16765555**	6
Cefditoren	Urinary Tract Infections	**20542206;8455334**	7
Cefditoren	Escherichia coli Infections	without evidence	8
Cefprozil	Urinary Tract Infections	**26391612**	9
Aztreonam	Soft Tissue Infections	without evidence	10

The entries in boldface represent the drug-disease associations supported by recent papers in PubMed but not included in CTD

**Table 6:** JI predicted drug-disease associations (top 10 in ranking list), and the corresponding evidence

Drug	Disease	Evidence (PMID)	Rank
Alprostadil	Tetralogy of Fallot	3543871	1
Alprostadil	Tricuspid Atresia	without evidence	2
Alprostadil	Hypoplastic Left Heart Syndrome	without evidence	3
Cefepime	Escherichia coli Infections	**26815433**	4
Cefepime	Urinary Tract Infections	**26243291;1804010**	5
Atorvastatin	Hypercholesterolemia	24593216;20946910;20135644	6
Cefotaxime	Escherichia coli Infections	without evidence	7
Clofibrate	Hypercholesterolemi	1175893;7080553;7157849	8
Fenofibrate	Hypercholesterolemi	2492189;24593216;2045526	9
Levofloxacin	Escherichia coli Infections	without evidence	10

The entries in boldface represent the drug-disease associations supported by recent papers in PubMed but not included in CTD

**Table 7:** HGBI predicted drug-disease associations (top 10 in ranking list), and the corresponding evidence

Drug	Disease	Evidence (PMID)	Rank
Lithium	Conduct Disorder	7491395;7691178;7751258	1
Lithium	Depressive Disorder	27752079;2723135;21252007	2
Ertapenem	Pyelonephritis	**22563210**	3
Moxifloxacin	Pyelonephritis	without evidence	4
Gatifloxacin	Pyelonephritis	**11911553;15037328**	5
Methotrexate	Psoriasis	20178709;19626273;19323665	6
Levofloxacin	Pyelonephritis	without evidence	7
Cefamandole	Staphylococcal Infections	9419181	8
Cefprozil	Staphylococcal Infections	without evidence	9
Vinblastine	Kidney Neoplasms	8602639;11194540	10

The entries in boldface represent the drug-disease associations supported by recent papers in PubMed but not included in CTD

**Table 8:** NBI predicted drug-disease associations (top 10 in ranking list), and the corresponding evidence

Drug	Disease	Evidence (PMID)	Rank
Flurandrenolide	Facial Dermatoses	without evidence	1
Levofloxacin	Urinary Tract Infections	**25931244**	2
Cefoperazone	Escherichia coli Infections	without evidence	3
Flurandrenolide	Scalp Dermatoses	without evidence	4
Ceftazidime	Proteus Infections	without evidence	5
Ceftizoxime	Escherichia coli Infections	**24755996**	6
Moxifloxacin	Streptococcal Infections	19188393;18818055;17562794	7
Cefpodoxime	Escherichia coli Infections	**23537823**	8
Ofloxacin	Streptococcal Infections	19856068	9
Ampicillin	Streptococcal Infections	2306432	10

The entries in boldface represent the drug-disease associations supported by recent papers in PubMed but not included in CTD

From Table 4, 5 out of top 10 predicted associations are supported by associations in CTD, the other two drug-disease pairs are supported by recent papers in PubMed but not include in CTD. From Table 5, we can clearly see that 1 out of top 10 predicted associations is supported by CTD and the other six associations are supported by recent papers in PubMed. From Table 6, JI totally finds 6 drug-disease pairs with evidence among the top 10 predicted associations. From Table 7, 5 out of top 10 predicted associations are supported by associations in CTD, the other two drug-disease pairs are supported by recent papers in PubMed but not include in CTD. From Table 8, NBI can find 6 associations with evidence. In summary, DR-IBRW can make more confident drug-disease repositioning than these comparing methods.

### Quantified individual walk length is reasonable

The drug-disease association prediction task is frequently modeled as a link prediction problem in a heterogeneous graph [36–38]. The link prediction relies on calculating the similarity between nodes. The number of paths between nodes and walk lengths are regarded as effective similarity metrics in the social network and biological network [36, 39, 40]. The similarities between drugs and diseases can be measured based on the number of walks that connect drug nodes and disease nodes in the network. Integrating the number of walks and their lengths can more comprehensively quantify the potential association probability of the drug-disease pair. In addition, the contribution of different nodes in the heterogeneous network is different. In other words, the information carried by each node in the heterogeneous work is imbalanced. Therefore, it is an issue to adopt a fixed walk-length for all nodes in link prediction.

**Fig. 5:**
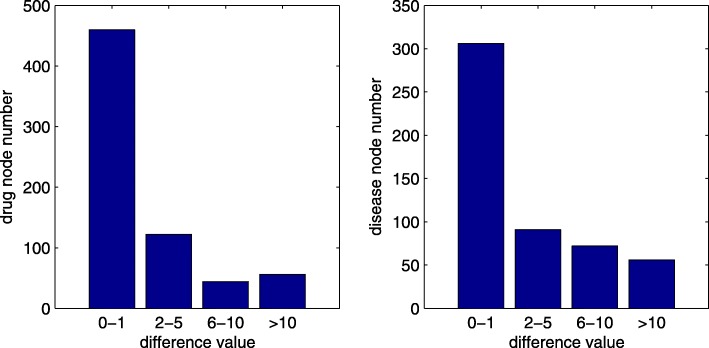
The margin between **P**_*r*_ and **L**_*r*_ for drugs (Left), and the margin between **P**_*d*_ and **L**_*d*_ for diseases (Right)

In order to answer why the choice of quantified individual walk length is reasonable, we calculate the shortest path for each drug and disease node, and measure the difference between shortest path and quantified individual walk length. We use the matrix **SP**(*r*_*i*_,*d*_*j*_) to represent the shortest path from the *i*−*th* drug to *j*−*th* disease, $\mathbf {SP} \in \mathbb {R}^{(n_{r}+n_{d}) \times (n_{r}+n_{d})}$. To calculate **SP**, we firstly construct an adjacency matrix **W**:

$$\mathbf{W}= \begin{bmatrix} \mathbf{W}_{rr} & \mathbf{W}_{rd} \\ \mathbf{W}_{dr} & \mathbf{W}_{dd} \end{bmatrix} $$

where $\mathbf {W}_{rr} \in \mathbb {R}^{n_{r} \times n_{r}}$ contains the shortest path between each two drug nodes, $\mathbf {W}_{dd} \in \mathbb {R}^{n_{d} \times n_{d}}$ contains the shortest path between each two disease nodes. **W**_*rd*_ is the drug-disease association matrix and **W**_*dr*_ is the transpose of **W**_*rd*_. Then, we adopt the Dijkstra algorithm to compute the shortest path between two nodes in matrix **W**. $\phantom {\dot {i}\!}\mathbf {P}_{r}=(rp_{1},rp_{2},\ldots,rp_{i},\ldots,{rp}_{n_{r}})$ where *rp*_*i*_ represents the longest path in the shortest path between *i*-th drug and all the diseases. $\phantom {\dot {i}\!}\mathbf {P}_{d}=(dp_{1},dp_{2},\ldots,dp_{j},\ldots,{dp}_{n_{d}})$ where *dp*_*j*_ represents the longest path in the shortest path between *j*-th disease and all the drugs. In other words, *rp*_*i*_ is the maximum shortest path for drug *i*, which can include nearly all the path information with diseases. *dp*_*j*_ is the maximum shortest path for disease *j* and it can approximately represent the path between disease *j* and all the drugs. **L**_*r*_ and **L**_*d*_ store the quantified individual walk-lengths of *n*_*r*_ drugs and *n*_*d*_ diseases. After that, we calculate the margin between **P**_*r*_ and **L**_*r*_ for drugs, and that between **P**_*d*_ and **L**_*d*_ for diseases. The statistical results are shown in Fig. 5. We can find that nearly 60% nodes’ differences are no larger than one. It can explain that the quantified individual walk lengths of most nodes are inline with the shortest path between the respective nodes. However, the maximum shortest path can only partially represent the path information from a drug node to a disease node. **L**_*r*_ can give more emphasize on shorter path between diseases and drugs than maximum shortest path, and it generally has a smaller value than **P**_*r*_. It is recognized that the shorter the distance between two nodes, the larger the similarity between them is. For these reasons, our random walk with individual walk achieves more prominent performance than random walk fixed walk length (as shown in Fig. 3)

**Fig. 6:**
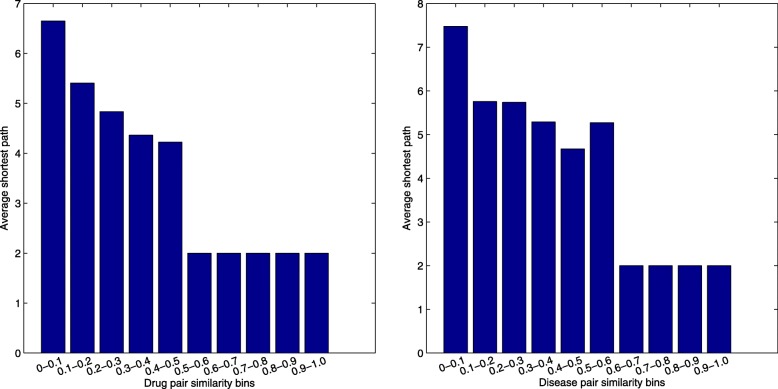
The average shortest path in different drug similarity subranges (Left), and in different disease similarity subranges (Right)

We also perform the correlation analysis on drug similarity matrix **S**_*r*_ and drug shortest path matrix **W**_*rr*_. We firstly partition **S**_*r*_ into ten subranges ((0, 0.1], (0.1, 0.2], etc.) and then partition **W**_*rr*_ into ten subranges to ensure that all the drug pairs in each subrange of **S**_*r*_ falling into the corresponding subrange of **W**_*rr*_. Next, we calculate the average shortest path of each subrange for **W**_*rr*_, and compute the correlation of average shortest paths and drug similarities between **W**_*rr*_ and **S**_*r*_ in each subrange. Similarly, we conduct the correlation analysis on disease similarity **S**_*d*_ and disease shortest path matrix **W**_*dd*_ in the same way and report the results in Fig. 6. We can clearly observe that the average shortest paths between drug pairs or disease pairs decrease as the increases of their similarities. This observation also differentiates the contribution of different walk lengths based on the assumption that nodes with shorter walk lengths contribute more to the similarity between two nodes.

## Conclusion

In this paper, we proposed a computational drug repositioning approach that encodes the drug chemical structure information, disease symptom information and known drug-disease interactions information into a heterogeneous network. Our approach accounts for structural difference of subnetworks of the heterogeneous network by bi-random walk, and for the contribution differences of different nodes via specifying quantified individual walk-lengths to them. Experimental study demonstrates that our approach performs better than other related competitive methods and the individual walk lengths contribute to an improved performance. We want to remark that our proposed approach can be easily generalized to predict links between nodes of a heterogeneous network.

## Data Availability

The datasets used during the current study are available from the corresponding author on reasonable request.

## Abbreviations

AUPR: The area under precision-recall curve
AUROC: The area under the receiver operating characteristic curve
CTD: Comparative Toxicogenomics Database
DR-IBRW: Drug repositioning approach based on individual bi-random walks
OMIM: Online Mendelian Inheritance in Man
PR: precision-recall
ROC: receiver operating characteristic
